# Old Masters of Medicine

**Published:** 1908-01-11

**Authors:** 


### The Evolution of Medicine.

### OLD MASTERS OF MEDICINE.

### I. CLAUDIUS GALENUS AND HIS SYSTEM.

Claudius Galenus, better known to English readers as Galen, was born in 131 A.D. at Pergamus. His connection with medical history is such that no one can afford to pass by his career without doing homage to his genius, or acknowledging the remarkable influence which his name and the theories he enunciated obtained in medicine. Those who believe that perverted truth, the half-caught glimmer, soon to be shrouded in the flimsy veils of sophistical argument and scholastic subtlety, the light that seen far off was too faint and evanescent to illuminate the whole range of medicine, and the experience that served only to lead astray, instead of to direct and guide; those who believe that these things set back human thought and the progress of medicine for so many centuries will have much to say in condemnation of the Pergamian. On the other hand, those who are willing to impute to the master a modicum of honesty, and to admit that he saw clearly in many points, however much his followers may have failed to grasp the significance of his observations, will acknowledge that the Galenic influence, so far as medicine was concerned, was not without benefit.

Galen's father was the architect Nicon, a man of more than ordinary acumen, well-read, wealthy, and prosperous. A follower of the Academicians himself, Nicon saw to it that his son obtained a sound education in literature, science, mathematics, and philosophy, and Galen is ever ready to speak with reverence and affection of the debt he owed his father—in striking contrast to the manner in which he alludes to his mother, who appears to have been a woman of light, if not loose, character. As a boy Galen was under the care of the Academician Cajus, who kept a school at Pergamus. Here he learned the old philosophy and gained a sound knowledge of Latin as well as Greek. He varied his studies by attending the periodical classes of the peripatetic teachers who from time to time visited and taught at Pergamus. Nicon, who knew the value of original thought and observation, instilled into the boy's mind a love for argument and contradiction. To prove the teachings of his master, to attempt to sift the false from the true, and to read largely and extensively on every subject were the ideals he set before his son, and it is probable that it was to this influence that Galen owed his broad-minded, unprejudiced acquaintance with the various philosophical theories of the day.

Our information concerning his boyhood is largely fragmentary and to a great extent wholly untrustworthy. His followers and disciples, who came to revere his memory with the reverence that admiration pays to genius, have encrusted the story of his life with a mass of traditional and legendary detail, much of which is palpably absurd, and little of which is necessary for a proper understanding of his character. A few facts, however, stand out in bold relief. As a boy he wrote a commentary on Aristotle; later on, while a medical student, he did a similar service for Theophrastus. Neither of these youthful works has been preserved, and we are therefore not in a position to judge of his skill as an infant prodigy. Nor are we certain as to what induced him to give up pure philosophy for medicine. Tradition tells that, influenced by a vision in which he saw his son warding off the attacks of death, Nikon persuaded him, while only sixteen, to attend the medical school, at that time a good one, which existed at Pergamus. Here the anatomist Satyrus demonstrated on the bony skeleton; the empiric Æschrion lectured on diagnosis and ætiology; Stratonicus, an old man imbued with the doctrines of the Hippocratic school, taught the principles of treatment according to the theories of the Dogmatists; and the renowned Ennius Meccius, reputed the best pharmacologist of the day, gave instruction in botany and therapeutics.

When, in 152, his father died, Galen found himself in possession of a modest competency, and determined to go further afield in search for knowledge. The fame of other schools in Mesopotamia, Egypt, and Arabia attracted him, and accordingly he pilgrimaged to Smyrna. Here the aged physician Pelops, the successor of the Academicians Quintus and Albinus, a man noted for his knowledge of comparative anatomy, lectured daily, and Galen enlisted in the throng of students that attended his demonstrations. From Smyrna the young Pergamian went to Corinth, where the anatomical instruction was more perfect than elsewhere. Not only wax and marble models (one of the latter of which is still to be seen in the Vatican Museum), but well prepared anatomical specimens were exhibited by the anatomist Numisianus. Besides, there was an opportunity, of which Galen eagerly availed himself, of actually dissecting human bodies. His first dissection was of a corpse which he found washed up on the river bank, half decomposed, but useful nevertheless. From Corinth he went to Alexandria, then declining as a school of learning. There was a time when this famous school had known better days. It had been the centre of intellectual thought and culture under the Ptolemies, and a remnant of its past glory still remained. The Museum still stood—that wonderful building in the Bruchium, which is said to have lodged more than 14,000 students and teachers, and the Serapion library (with its 300,000 odd volumes) was still available for research. Probably the botanical garden and the nucleus of a zoological garden, founded by Philadelphus, were also still in existence. But the medical school, which under Erasistratus and Herophilus had reached the summit of its greatness, was no longer the acknowledged premier academy of medicine. Heraklianus was there, it is true, and under him Galen studied for a time. It is only reasonable to suppose that the young student, in his zealous search for truth, made ample use of the magnificent library.

*(To be continued.)*

